# Complete Coding Sequences of Three Toscana Virus Strains Isolated from Sandflies in France

**DOI:** 10.1128/genomeA.01676-15

**Published:** 2016-02-11

**Authors:** Amal Baklouti, Isabelle Leparc Goffard, Geraldine Piorkowski, Bruno Coutard, Nicolas Papageorgiou, Xavier De Lamballerie, Rémi N. Charrel

**Affiliations:** a EPV Emergence des Pathologies Virales Aix Marseille Université, UMR_D 190 IRD French Institute of Research for Development, U1207 INSERM, EHESP French School of Public Health, Marseille, France; b IHU Méditerranée Infection, APHM Public Hospitals of Marseille, Marseille, France; c Institut de Recherche Biomédicale des Armées, National Reference Laboratory for Arboviruses, Marseille, France; d Aix Marseille Université/CNRS, AFMB UMR 7257, Marseille, France

## Abstract

Toscana virus (TOSV) is an arthropod-borne virus belonging to the sandfly fever Naples virus species within the genus *Phlebovirus*. We report here the complete coding sequences of three TOSV strains belonging to lineage B and isolated from sandflies trapped in the Southeast of France between 2009 and 2013.

## GENOME ANNOUNCEMENT

Toscana virus (TOSV) belongs to the species sandfly fever Naples virus (family *Bunyaviridae*, genus *Phlebovirus*) (1). TOSV is an arthropod-borne virus transmitted by phlebotomine flies in the Old World. TOSV is an enveloped virus with a single-strand negative-sense RNA genome, which consists of three segments (large [L], medium [M], and small [S]). L encodes the viral polymerase, M encodes Gn and Gc glycoproteins and the NSm nonstructural protein, and S encodes the nonstructural (NS) protein and the nucleoprotein (N) (2, 3).

TOSV was first isolated in 1971 from *Phlebotomus perniciosus* in central Italy (1). Other strains were later isolated in Italy and other western Mediterranean countries (Portugal, Spain, and France) (4, 5), as well as in the eastern Mediterranean area (Greece, Croatia, and Turkey) and in northern Africa (Tunisia, Morocco, and Algeria) (6–8).

From April to October, TOSV can cause febrile illness and is also a major cause of central and peripheral nervous system manifestations. Two genotypes (or lineages) exist that are more or less geographically driven (4). Lineage A strains have been identified in Italy, France, Tunisia, Algeria, and Turkey (5–7), and lineage B strains have been detected in Spain, Portugal, France, Morocco, and Turkey (8, 9). France and Turkey are the only countries where the two lineages cocirculate. Recently, a third lineage was identified genetically in Croatia and Greece, although virus isolation was not done (10, 11).

Despite the geographic area where TOSV is present covering immense territories, only four full-length sequences were available at the outset of this study: three for lineage A strains from Italy, Tunisia, and Algeria, and one for a lineage B strain (France).

We determined the complete coding sequences of three strains (TOSV-P51, TOSV-P233, and TOSV-113) isolated from *P. perniciosus*. Viral RNA extracted from a cell culture supernatant at passage 3 was used for next-generation sequencing using PGM Ion Torrent (Life Technologies) after nonspecific amplification. Reads were mapped on reference sequences to produce long consensus contigs. Parameters were set such that each accepted read had to map to the reference sequence for at least 50% of its length, with a minimum of 80% identity to the reference (accession no. EF656361 to EF656363). For TOSV-P233, 16,321 of a total 176,394 reads matched with the reference sequence; for TOSV-P51, 70,601 of a total of 183,763 reads matched the reference; and for TOSV-113, 63,305 of a total of 192,749 reads matched the reference. PCR was performed to fill the remaining gaps between contigs, and sequencing was performed using the Sanger method.

The open reading frames (ORFs) of the 3 strains encoded N (254 amino acids [aa]), NSs (316 aa), Gn (527 aa), Gc (502 aa), and L (2,101 aa).

The full-length sequences of each gene were aligned with homologous TOSV sequences using the Clustal algorithm. Neighbor-joining analysis (Kimura 2-parameter and *p*-distance models) was performed by MEGA 6, with 1,000 bootstrap pseudoreplications. Phylogenetic analysis indicated that the three strains belong to lineage B. These 3 TOSV strains are accessible for academic research in the European Virus Archive (EVAg).

### Nucleotide sequence accession numbers.

The sequences of TOSV-P233 (accession numbers KU204975 to KU204977), TOSV-P51 (accession numbers KU204978 to KU204980), and TOSV-113 (accession numbers KU204981 to KU204983) were deposited in GenBank.
